# Resistance to antibacterial antifolates in multidrug-resistant *Staphylococcus aureus*: prevalence estimates and genetic basis

**DOI:** 10.1093/jac/dkad063

**Published:** 2023-03-20

**Authors:** Louise Kime, Tina Waring, Merianne Mohamad, Benjamin F Mann, Alex J O’Neill

**Affiliations:** School of Molecular and Cellular Biology, Faculty of Biological Sciences, University of Leeds, Leeds LS2 9JT, UK; School of Molecular and Cellular Biology, Faculty of Biological Sciences, University of Leeds, Leeds LS2 9JT, UK; School of Molecular and Cellular Biology, Faculty of Biological Sciences, University of Leeds, Leeds LS2 9JT, UK; School of Molecular and Cellular Biology, Faculty of Biological Sciences, University of Leeds, Leeds LS2 9JT, UK; School of Molecular and Cellular Biology, Faculty of Biological Sciences, University of Leeds, Leeds LS2 9JT, UK

## Abstract

**Objectives:**

Antibacterial antifolate drugs might have a wider role in the management of staphylococcal infection. One factor that could potentially limit their use in this context is pre-existing resistance. Here we explored the prevalence and genetic basis for resistance to these drugs in a large collection (*n *= 1470) of multidrug-resistant (MDR) *Staphylococcus aureus*.

**Methods:**

Strains were subjected to susceptibility testing to detect resistance to trimethoprim, sulfamethoxazole, co-trimoxazole and the investigational drug, iclaprim. Whole-genome sequences were interrogated to establish the genetic basis for resistance.

**Results:**

According to CLSI breakpoints, 15.2% of the strains were resistant to trimethoprim, 5.2% to sulfamethoxazole and 4.1% to co-trimoxazole. Using the proposed breakpoint for iclaprim, 89% of the trimethoprim-resistant strains exhibited non-susceptibility to this agent. Sulfamethozaxole resistance was exclusively the result of mutation in the drug target (dihydropteroate synthase). Resistance to trimethoprim and iclaprim also resulted from mutation in the target (dihydrofolate reductase; DHFR) but was more commonly associated with horizontal acquisition of genes encoding drug-insensitive DHFR proteins. Among the latter, we identified a novel gene (*dfrL*) encoding a DHFR with ∼35% identity to native and known resistant DHFRs, which was confirmed via molecular cloning to mediate high-level resistance.

**Conclusions:**

This study provides a detailed picture of the genotypes underlying staphylococcal resistance to antifolate drugs in clinical use and in development. Prevalence estimates suggest that resistance to the diaminopyrimidines (trimethoprim/iclaprim) is not uncommon among MDR *S. aureus*, and considerably higher than observed for sulfamethoxazole or co-trimoxazole.

## Introduction

*Staphylococcus aureus* is an important human pathogen whose impact has been exacerbated by its ability to evolve resistance to antibiotics, a phenomenon exemplified by the emergence of methicillin-resistant *S. aureus* (MRSA).^1^ Vancomycin remains the first-line treatment for MRSA infection in many contexts, though its performance is typically inferior to that of the β-lactams and resistance has emerged in recent decades.^1–3^ Although newer drugs have been developed that are active against multidrug-resistant (MDR) *S. aureus*, these typically have their own drawbacks, such as the requirement for parenteral administration, adverse side effects, the development of resistance and expense.^1, 4^ Consequently, there remains a need for additional therapeutic options. One potential source of these might be antibacterial agents that are at present little used in treating staphylococcal infection, such as the antifolate drugs.^5, 6^

Antibacterial antifolate drugs fall into two classes; the sulfa drugs (exemplified by sulfamethoxazole) and the diaminopyrimidines, the only licensed representative of which is trimethoprim. Both classes act to prevent bacterial biosynthesis of tetrahydrofolate, a co-factor essential for the *de novo* synthesis of thymidine.^7^ However, they act on distinct enzyme targets within the tetrahydrofolate biosynthesis pathway to achieve this end, with the sulfa drugs targeting dihydropteroate synthase (DHPS) and the diaminopyrimidines inhibiting dihydrofolate reductase (DHFR). The action of the two classes together is highly synergistic,^8^ a phenomenon that has been exploited in the combination drug, co-trimoxazole (a mixture of trimethoprim and sulfamethoxazole). A potential limitation of antifolates for antistaphylococcal chemotherapy is that the metabolic block they effect can be bypassed by bacterial uptake of exogenous thymidine from damaged tissues, thereby reducing clinical efficacy.^9^ Consequently, they are typically indicated for staphylococcal infections associated with low bacterial burden,^10^ including skin and soft tissue infection and uncomplicated urinary tract infection.^11–13^ Nevertheless, some studies suggest that co-trimoxazole might have comparable efficacy to vancomycin in the treatment of MRSA bacteraemia.^14, 15^

In addition to the licensed antifolates described above, an investigational diaminopyrimidine (iclaprim) has reached an advanced stage of clinical evaluation. This agent was designed to exhibit greater antibacterial potency than trimethoprim and to retain activity in the face of common trimethoprim resistance mechanisms.^16, 17^ Iclaprim demonstrates potent activity against MRSA,^18–20^ and two phase 3 clinical trials (REVIVE-1 and REVIVE-2) deemed it non-inferior to vancomycin in the treatment of acute bacterial skin and skin structure infections.^21, 22^ However, iclaprim has yet to be approved by either the EMA or the FDA.

Since representatives of the sulfa drugs and diaminopyrimidines have been in clinical use for many decades—both alone and in combination—key factors in assessing their potential use for wider deployment as antistaphylococcal agents are the prevalence of pre-existing resistance and whether such resistance has the potential to spread through horizontal gene transfer. As briefly outlined next, while some information already exists regarding the nature and prevalence of antifolate resistance in *S. aureus*, a detailed picture is currently lacking.

Two types of trimethoprim resistance have been described in *S. aureus*: (i) mutational resistance in the chromosomal *dfrB* gene encoding DHFR, resulting in amino acid substitutions that render the protein less susceptible to the action of trimethoprim and (ii) the horizontal acquisition of genes encoding trimethoprim-insensitive variants of DHFR.^16, 23–27^ By contrast, there have been no reports of horizontally acquired resistance to sulfamethoxazole in *S. aureus*, with resistance instead attributed to mutational change in the *folP* gene that encodes DHPS.^28^ A number of publications have examined the prevalence and nature of antifolate resistance in clinical isolates of *S. aureus*, although these studies typically have a narrow focus in respect of the antifolate agents studied (usually co-trimoxazole only) or the geographical location from which isolates were recovered, and are limited in the extent to which the genetics of resistance has been interrogated. The prevalence of resistance to co-trimoxazole is typically lower in the global north;^18, 29–32^ rates of resistance are higher in the global south where this combination drug is used as a first-line treatment^33^ and is also employed as prophylaxis for *Pneumocystis* infections in HIV patients.^34^ Three studies have explored susceptibility among clinical isolates not only to co-trimoxazole but also to one or both of its constituent drugs, and determined the genetic basis of resistance to trimethoprim alone.^35–37^ A more recent study assessed susceptibility to co-trimoxazole only, and examined the basis of resistance to the constituent drugs in a small subset of the isolates.^38^ At present there is only limited information available regarding the nature of resistance to iclaprim in *S. aureus*.^39, 40^

With a view to gaining a more complete understanding of antifolate resistance in *S. aureus*, we have determined the susceptibility of a large, diverse collection (*n *= 1470) of clinical isolates to all clinically deployed antifolate classes and iclaprim, and comprehensively defined the genetic basis of resistance. In view of the overarching aim of this work to assess to what extent antifolates might have a wider role in the treatment of infections caused by *S. aureu*s resistant to commonly deployed antistaphylococcal agents, this analysis used a collection of MDR strains.

## Materials and methods

### Bacteria and susceptibility testing

The collection of *S. aureus* isolates (*n = *1470) employed here was described in a recent study,^41^ and comprises strains from mainland Europe (*n = *556; 37.8%), UK (*n = *524; 35.6%), USA (*n = *192; 13.1%), Asia (*n = *101; 6.9%), Oceania (*n = *12; 0.8%), Latin America (*n = *2; 0.1%) and some of unknown geographical origin (*n = *83; 5.6%). *S. aureus* SH1000^42^ was used as an antibiotic-susceptible control organism. Susceptibility testing was performed by agar dilution,^43^ using cation-adjusted Mueller–Hinton broth and agar (Becton Dickinson). Trimethoprim and sulfamethoxazole (Sigma-Aldrich) were used independently for susceptibility testing and were also combined in a 1:19 ratio^44^ for co-trimoxazole susceptibility testing. Where available, both EUCAST and CLSI breakpoints were used to interpret susceptibility data.^44, 45^ No confirmed resistance breakpoints exist for iclaprim (MedChem Express), but an iclaprim MIC of ≥2 mg/L for *S. aureus* has been proposed to denote non-susceptibility.^46^

### Characterization of resistance genotypes

Whole-genome sequences were interrogated for known acquired trimethoprim resistance determinants in *S. aureus* using ARIBA,^47^ employing reference sequences for *dfrA* (GU565967), *dfrG* (AB205645) and *dfrK* (FM207105). TBLASTN^48^ was used to detect newly acquired *dfr* variants and to identify mutational resistance in the chromosomal genes encoding DHFR (*dfrB*) and DHPS (*folP*). Staphylococcal DHPS is known to exhibit considerable natural polymorphism;^28,49^ care was therefore taken in our analysis to disregard amino acid variation with no apparent role in resistance.

Putative trimethoprim resistance determinants were further investigated by assessing their ability to confer resistance when introduced into an antibiotic-susceptible strain. The *dfrB* gene (encoding wild-type DHFR) and a *dfrB* variant encoding amino acid substitution G_73_D were PCR amplified with oligonucleotide primers 5′-GGAAAGGATCCACTATGAATCACATCCAGC and 5′-CTGGGATCCTAATCTGTTTTGTCATGGTCG (engineered restriction sites underlined), while the *dfrL* gene was amplified using 5′-GGAAAGGATCCTGGAATGGGATTCAGGAGTGG (primer F1) and 5′-CTGGGATCCCTGGCGAAGTCCAATCTGGT (primer R1). The latter gene was also amplified together with the upstream gene, *ant4*, using 5′-GGAAAGGATCCCCCAGTTTGTACTCGCAGGT (primer F2) and R1, and *ant4* was amplified alone (as a control) using F2 and 5′-CTGGGATCCGCGATTGGTGCTCTGATTCC (primer R2). The resulting amplicons were ligated into plasmid pCU1^50^ at the *Bam*HI site, propagated in *E. coli*, and introduced into *S. aureus* RN4220^51^ by electroporation.

## Results

### Prevalence estimates for resistance to antifolate drugs

The clinical strains of *S. aureus* used in this study are all MDR, defined in this case as resistant to at least two clinically deployed antistaphylococcal drug classes.^41^ The entire collection was initially screened on agar for resistance to trimethoprim, sulfamethoxazole and iclaprim using concentrations around the established or proposed breakpoint values; strains showing resistance to any of these agents were subsequently also tested for resistance to co-trimoxazole. The genomes of strains resistant to at least one of these agents were interrogated to identify relevant resistance mechanisms, and representative strains for each independent resistance genotype were subjected to full susceptibility testing to determine precise MIC values (Table 1). We noted differences in the published breakpoints for resistance to some of the drugs between the two major bodies that set breakpoints (EUCAST and CLSI), and therefore considered both values when reporting resistance prevalence below.

**Table 1.** Representative examples of all trimethoprim, sulfamethoxazole, co-trimoxazole and iclaprim resistance genotypes identified in this study, and their associated resistance phenotypes

				MIC (mg/L)			
Representative strain	ENA accession number	DHFR mutation/variant^a^	DHPS mutation	Trimethoprim	Sulfamethoxazole	Co-trimoxazole	Iclaprim
MOS236	ERS1531789	*dfr*A + *dfr*G	—	>2048	64	2	>32
DUB20	ERS1452229	*dfr*K	—	>2048	32	0.5	>32
MOS20	ERS1531558	*dfr*G	—	2048	32	0.5	>32
MOS222	ERS1531775	*dfrL*	—	1024	32	0.5	>32
MOS43	ERS1531581	*dfr*A	—	512	32	0.5	32
MOS73	ERS1531611	F_99_Y	—	64	32	0.25	2
MOS266	ERS1531819	L_25_I, L_41_F	—	64	8	0.25	2
NRS844	ERS1580709	H_150_R	—	32	32	1	4
A75	ERS1452203	L_41_F	—	16	32	0.5	0.5
NW84	ERS1179738	F_99_Y, V_113_I, A_135_T	—	64	16	0.25	4
MOS251	ERS1531804	F_99_Y, A_135_T	—	32	4	0.125	2
MOS261	ERS1531814	A_135_T, H_150_R	—	16	≤2	0.125	1
RVC29	ERS1452260	L_41_F, A_135_T	—	16	16	0.25	0.5
MOS113	ERS1531651	—	T_51_M, E_208_K	2	1024	1	ND
NRS715	ERS1179826	—	F_17_L, A_184_V	1	512	0.5	ND
MOS89	ERS1531627	—	F_17_L, KE_257__dup	1	512	0.5	ND
Y57	ERS1452124	*dfr*G	F_17_L, A_184_V	>2048	>2048	64	>32
NRS27	ERS1179781	*dfr*A	F_17_L, A_184_V	>2048	>2048	64	>32
NRS685	ERS1580583	*dfr*G	F_17_L, E_208_K	>2048	2048	32	>32
MOS234	ERS1531787	*dfr*G	F_17_L, KE_257__dup	>2048	1024	32	>32
DUB21	ERS1452230	*dfr*A	F_17_L, E_208_K	1024	1024	16	>32
DUB25	ERS1452234	F_99_Y, H_150_R	F_17_L, KE_257__dup	512	1024	16	32
MOS88	ERS1531626	*dfr*A	F_17_L, KE_257__dup	512	1024	8	>32
R38	ERS1179860	L_21_V, N_60_I, F_99_Y	F_17_L, KE_257__dup	256	512	8	16
R37	ERS1179859	*dfrA* + L_21_V, N_60_I, F_99_Y	F_17_L, KE_257__dup	ND	ND	ND	ND
MOS5	ERS1531543	F_99_Y	F_17_L, KE_257__dup	32	2048	4	1
R39	ERS1179861	F_99_Y	T_51_M, E_208_K	64	512	2	2–4

ND:not determined, dup: duplication

DHFR substitutions V_113_I and A_135_T were found in strains carrying known trimethoprim resistance mutations and do not seem to contribute to trimethoprim resistance. However, A_135_T in combination with certain resistance mutations in DHFR seems to increase susceptibility to sulfamethoxazole; trimethoprim resistance genotypes containing this mutation have been grouped together in the table to highlight this difference.

**Figure 1.**
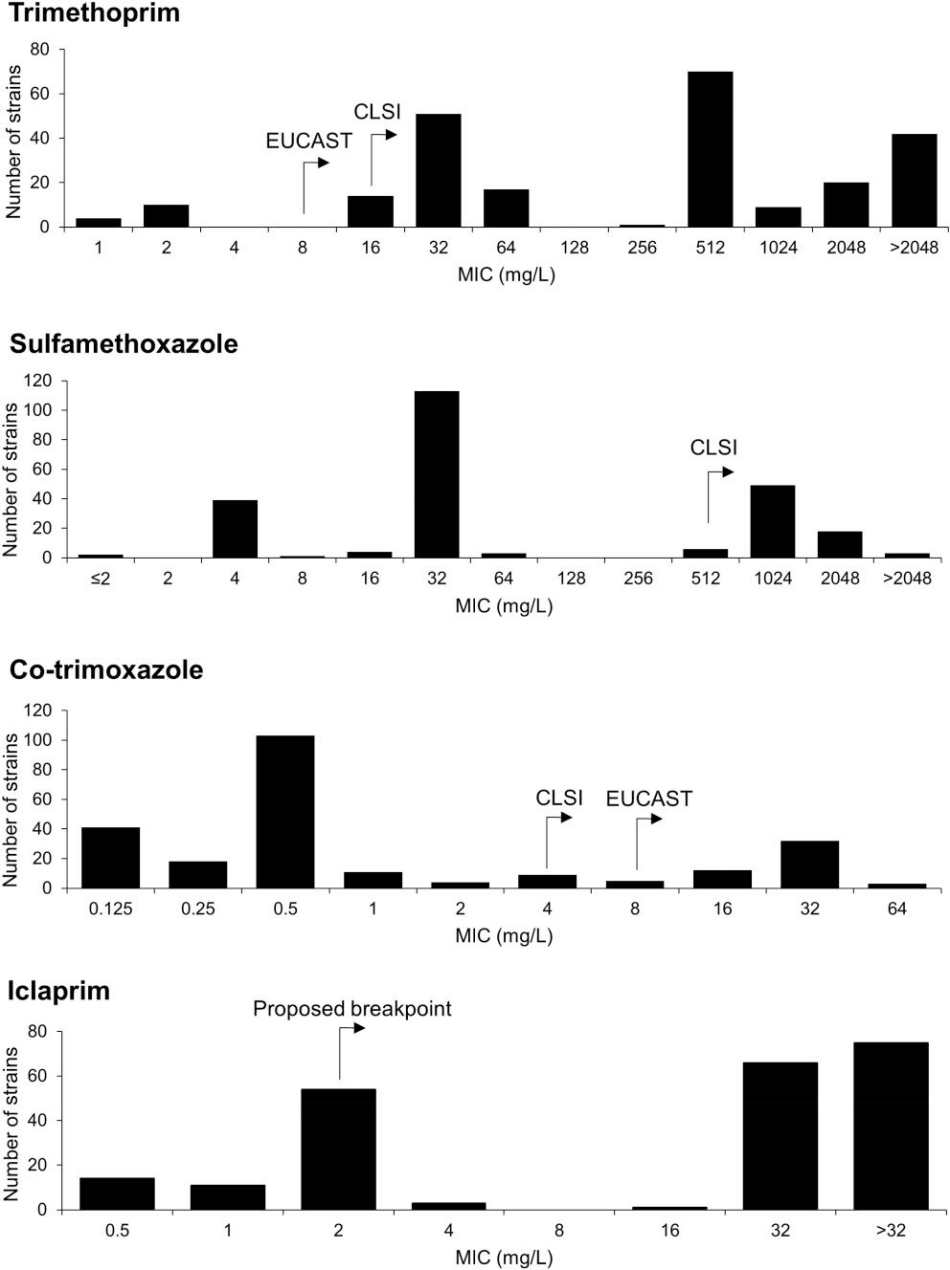
Distribution of MIC values for *S. aureus* strains resistant to antifolate drugs. The distribution of MIC values of all strains resistant to at least one drug (*n = *238) for trimethoprim, sulfamethoxazole and co-trimoxazole. MIC distribution for iclaprim was for those strains exhibiting trimethoprim resistance only (*n = *224). Clinical resistance breakpoint values set by CLSI and EUCAST, and the proposed breakpoint for non-susceptibility to iclaprim, are indicated with arrows.

Although the breakpoint values for trimethoprim resistance differ between EUCAST (R ≥ 8 mg/L) and CLSI (R ≥ 16 mg/L), 15.2% (*n = *224) of strains were judged to be resistant according to either criterion (since no strain was associated with an MIC of 8 mg/L) (Figure 1). For sulfamethoxazole, only CLSI publishes breakpoints for *S. aureus* (R ≥ 512 mg/L), and based on that value, 5.2% (*n = *76) of the strains were resistant (Figure 1). According to the CLSI breakpoint for co-trimoxazole (R ≥ 4 mg/L), 4.1% (*n = *61) of the strains were resistant; this figure decreased to 3.5% (*n = *52) when applying the EUCAST breakpoint (R ≥ 8 mg/L) (Figure 1). This discrepancy is explored further below. According to the proposed non-susceptibility breakpoint for iclaprim (≥2 mg/L), 13.5% (*n = *199) of the strains were non-susceptible to this agent (Figure 1). Extensive cross-resistance was observed between iclaprim and trimethoprim; all the iclaprim non-susceptible strains were trimethoprim resistant, while ∼89% (199 of 224) of trimethoprim-resistant strains exhibited non-susceptibility to iclaprim.

### Genetic basis for resistance to trimethoprim

**Figure 2.**
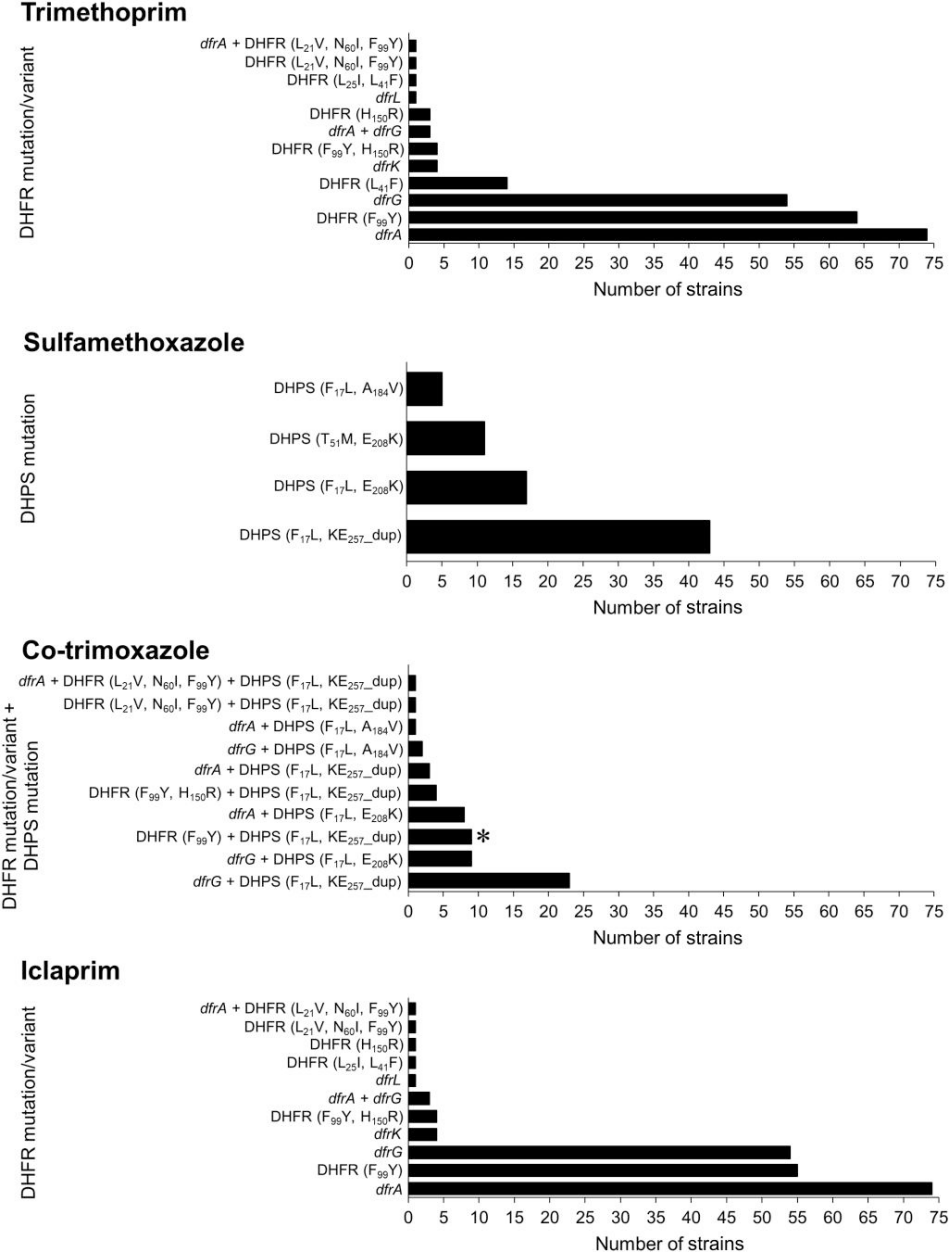
Relative prevalence of antifolate resistance genotypes in *S. aureus*. The charts show a tally for each independent resistance genotype in strains exhibiting resistance to trimethoprim (*n = *224), sulfamethoxazole (*n = *76), co-trimoxazole (*n = *61) or non-susceptibility to iclaprim (*n = *199). An asterisk (*) indicates the genotype that is resistant to co-trimoxazole according to CLSI only.

We identified 12 independent genotypes conferring trimethoprim resistance (Table 1, Figure 2). Of these, the horizontally acquired, trimethoprim-insensitive DHFR genes accounted for resistance in most of the resistant strains (137 of 224) and were invariably associated with high-level resistance (MIC of >256 mg/L). There was considerable variation in the prevalence of such DHFR determinants; *dfrA* and *dfrG* were both common (detected in 75 and 54 strains, respectively, and concurrently in three strains), while *dfrK* was not (*n = *4). Mutational resistance to trimethoprim resulting from genetic change in the native *dfrB* gene was detected in 88 of the 224 resistant strains and was typically associated with low-level resistance (≤64 mg/L); however, two *dfrB* genotypes conferred high-level resistance (DHFR substitutions F_99_Y/H_150_R and L_21_V/N_60_I/F_99_Y) (Figure 2, Table 1). Only a single strain (R37) harboured resistance mutations in DHFR (L_21_V/N_60_I/F_99_Y) while concurrently carrying an acquired trimethoprim resistance determinant (*dfrA*) (Table 1). The most common trimethoprim resistance mutation, encoding DHFR substitution F_99_Y, was found in most (70 of 88) strains exhibiting mutational resistance, usually in the absence of other resistance mutations. Most resistance genotypes in *dfrB* that we identified have been described previously.^16, 23, 27, 52, 53^ However, we detected an apparently novel mutation in strain MOS266, leading to DHFR substitution L_25_I, that appeared to increase by 4-fold the level of trimethoprim resistance associated with the L_41_F substitution also present in that strain; the trimethoprim MIC against MOS266 (L_25_I/L_41_F) was 64 mg/L compared to the MIC of 16 mg/L against strains carrying L_41_F alone (e.g. A75) (Table 1).

**Figure 3.**
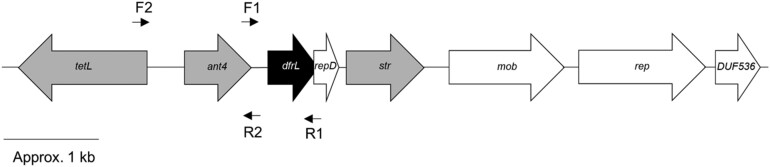
Genetic architecture of the plasmid harbouring *dfrL*. The *dfrL* gene (black) resides on an ∼8 kb plasmid, pSaMOS222_1, that also contains several other resistance determinants (grey); *tetL* (tetracycline resistance), *ant4* (aminoglycoside resistance) and *str* (streptomycin resistance). The plasmid also encodes a mobilization protein (*mob*), a protein containing a domain of unknown function (*DUF536*), a plasmid replication protein (*rep*) and a truncated protein corresponding to the C-terminal portion of the RepD replication initiator (*repD*). The positions of oligonucleotide primers used for amplification/cloning of *dfrL* are indicated.

In one strain (MOS222), no previously defined mechanism could be detected to account for the observed high-level trimethoprim resistance (MIC of 1024 mg/L). However, two potential sources of resistance were identified *in silico*. The nucleotide sequence of the native *dfrB* gene harbours a missense mutation encoding a G_73_D substitution, and TBLASTN analysis of the whole-genome sequence using *S. aureus* DHFR as the query returned a hit exhibiting ∼35% identity to both *dfrB*-encoded DHFR and the acquired, trimethoprim-resistant DHFR variants encoded by *dfrA*, *dfrG* and *dfrK*. The gene encoding this DHFR variant appeared to be located on an ∼8 kb multidrug resistance plasmid [designated pSaMOS222_1 (GenBank Accession CAIIKR010000012); Figure 3] and was provisionally named *dfrL* (GenBank Accession CAC8536249). To assess whether the mutant *dfrB* and/or the *dfrL* gene contribute to trimethoprim resistance in MOS222, we independently introduced each of these genes into a trimethoprim-susceptible *S. aureus* strain (RN4220). We have previously observed that introducing wild-type *dfrB* into a trimethoprim-susceptible host on a multicopy plasmid will itself cause a reduction in trimethoprim susceptibility, presumably as a consequence of increased gene dosage of the drug target;^27^ hence, we included such a construct here to control for this effect. Transformation with pCU1:*dfrB*_wt_ resulted in an 8-fold reduction in trimethoprim susceptibility compared to RN4220 (pCU1), raising the MIC from 4 mg/L to 32 mg/L. This same level of reduction in susceptibility was observed with a pCU1 construct encoding DHFR_G73D_, implying that this substitution does not mediate reduced susceptibility to trimethoprim. Transformation of a pCU1:*dfrL* construct into RN4220 also failed to reduce trimethoprim susceptibility above that observed in the strain carrying pCU1:*dfrB*_wt_. However, we speculated that this construct had incompletely captured the necessary promoter elements for full expression of *dfrL*; on the basis that the promoter driving expression of *dfrL* lies 5′ to the upstream gene (the aminoglycoside resistance gene, *ant4*) (Figure 3), we generated a further construct encompassing both genes and the likely promoter region. This latter construct conferred a 256-fold reduction in trimethoprim susceptibility (MIC of 1024 mg/L) compared to RN4220 (pCU1). To confirm that *dfrL* is alone responsible for this effect (i.e. that the *ant4* gene makes no contribution to trimethoprim resistance), we generated an otherwise-identical construct lacking *dfrL*; as anticipated, this construct conferred no reduction in trimethoprim susceptibility on RN4220. To confirm the apparent plasmid location of *dfrL*, purified plasmid from strain MOS222 was used to transform RN4220, with subsequent selection on agar with trimethoprim at 256 mg/L. Transformants were recovered that not only exhibited high-level trimethoprim resistance (MIC of >2048 mg/L), but concurrently demonstrated resistance to the other antibiotics for which pSaMOS222_1 harbours resistance determinants (tetracycline, tobramycin and streptomycin) (data not shown).

### Genetic basis for resistance to sulfamethoxazole

Sulfamethoxazole resistance was in all cases attributable to mutations in the gene encoding DHPS (*folP*), and only four independent resistance genotypes were identified (Table 1, Figure 2). All four genotypes involved two mutations in DHPS, with the DHPS_F17L, KE257_dup_ double mutation found in over half (43 of 76) of resistant isolates. In five strains, we detected an apparently novel resistance substitution in DHPS in the form of A_184_V, which was present in each case in combination with F_17_L. The role of this substitution in resistance is implied by the fact that the level of sulfamethoxazole resistance of strains harbouring F_17_L/A_184_V (Table 1) is considerably greater than that previously reported for a strain engineered to carry F_17_L alone.^28^

### Genetic basis for resistance to co-trimoxazole

Ten independent genotypes conferred co-trimoxazole resistance (Table 1, Figure 2). Resistance to co-trimoxazole typically involved carriage of *dfrA* or *dfrG* (seen in 47 of 52 resistant strains), often in conjunction with DHPS_F17L, KE257_dup_ (27 of 47 strains). The presence of a resistance determinant to one drug of the combination appeared in some instances to alter susceptibility to the other. In the absence of DHPS substitutions, *dfrA* is associated with a trimethoprim MIC of 512 mg/L (strain MOS43), while in the presence of the DHPS_F17L, A184V_ double mutation, the trimethoprim MIC associated with *dfrA* increased to >2048 mg/L (strain NRS27). Similarly, the sulfamethoxazole MIC associated with DHPS_F17L, A184V_ increased from 512 mg/L (NRS715) to >2048 mg/L (Y57 and NRS27) in the presence of either *dfrA* or *dfrG* (Table 1).

As anticipated, most of the strains independently resistant to both trimethoprim and sulfamethoxazole were also found to be resistant to co-trimoxazole, however, 10 such strains were not according to one or both of the CLSI/EUCAST breakpoints. Strain R39 (DHFR_F99Y_, DHPS_T51M, E208K_) was associated with a co-trimoxazole MIC of 2 mg/L, which is susceptible according to both EUCAST and CLSI (Table 1). The other nine strains, all carrying the same co-trimoxazole resistance genotype (DHFR_F99Y_, DHPS_F17L, KE257_dup_), were associated with an MIC of 4 mg/L (Table 1); this is resistant according to CLSI (R ≥ 4 mg/L), but not EUCAST (R ≥ 8 mg/L). Conversely, we also found one strain (SG171) that, while harbouring *dfrA*, would have been predicted to be co-trimoxazole susceptible based on its apparent susceptibility to sulfamethoxazole (MIC of 128 mg/L); however, it exhibited co-trimoxazole resistance according to CLSI guidelines (MIC of 4 mg/L).

### Genetic basis for non-susceptibility to iclaprim

We identified 11 independent genotypes responsible for non-susceptibility to iclaprim (Table 1, Figure 2). Of the strains resistant to trimethoprim, 89% (199 of 224) showed non-susceptibility to iclaprim, and no instances of iclaprim non-susceptibility were found in the absence of trimethoprim resistance. Higher-level resistance to iclaprim was most commonly mediated by acquisition of a *dfr* variant, including the *dfrL* gene described before, but was also associated with mutations within the native *dfrB* gene (strains harbouring the DHFR substitutions F_99_Y/H_150_R or L_21_V/N_60_I/F_99_Y) (Table 1, Figure 2). As for trimethoprim, lower-level resistance was the result of mutational change in *dfrB*.

## Discussion

This study has analysed resistance to several antifolate drugs in clinical use and/or in development against *S. aureus*, assessing both the prevalence and genetic basis of resistance in a large collection of strains, and thereby providing fresh insight into the nature of antifolate resistance in this organism.

The *S. aureus* strains used in this study were assembled from diverse clinical and geographical settings and are universally MDR, with most (∼86%) being MRSA. Given the nature of this collection, we recognize that the resulting resistance prevalence estimates are no substitute for those gained through formal surveillance. Nevertheless, they offer a unique perspective, providing an indication of resistance rates in a population of staphylococcal strains against which antifolates are most likely to be used (i.e. where resistance precludes the use of typical first-line treatments). For resistance to sulfamethoxazole and co-trimoxazole, prevalence was low (≤5% of strains tested); while there is little in the way of prevalence estimates for staphylococcal resistance to the former in the literature, the co-trimoxazole data accord with other recent studies.^18, 29–32^ Resistance rates to trimethoprim were considerably higher (∼15% of strain), a theme also seen in the handful of other studies that have analysed resistance prevalence to more than one antifolate drug.^36, 37^ Most of the trimethoprim-resistant strains (89%) we identified exhibited non-susceptibility to iclaprim, an overall prevalence rate of ∼13.5% iclaprim non-susceptibility in this study. Two independent surveillance studies have reported the prevalence of iclaprim non-susceptibility to be 4.6% and 6.7%.^19, 20^ Higher-level resistance to iclaprim was due, in most cases, to acquisition of a *dfr* variant, which is in accord with other recent studies.^39, 40^ Such resistance determinants were considered rare by the original developers of iclaprim;^16, 54^ the available data, including that presented here, suggest that these are more prevalent than originally supposed. Indeed, the high prevalence of *dfr* variants—and the existence of reportedly not uncommon and apparently distinct high-level iclaprim resistance mechanisms that do not give concurrent resistance to trimethoprim^20^—could represent obstacles to the successful deployment of iclaprim for the treatment of staphylococcal infection.

**Figure 4.**
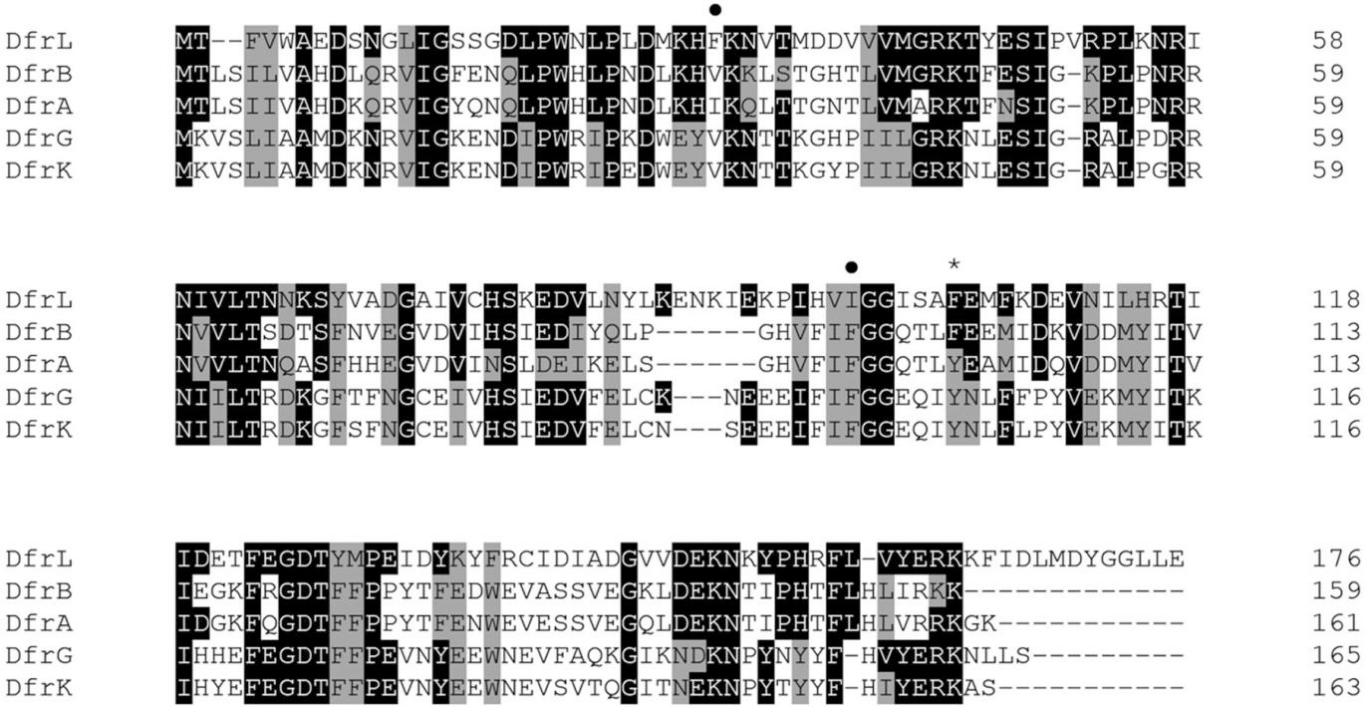
Amino acid sequence alignment of DfrL with other staphylococcal DHFRs. The protein sequence of DfrL was aligned using Clustal Omega with the native, trimethoprim-susceptible DHFR (DfrB) and the trimethoprim-resistant DHFRs encoded by *dfrA*, *dfrG* and *dfrK*. Bioinformatic analysis of the nucleotide region encoding *dfrL* revealed two potential translation start sites; in this figure, we have shown the protein whose predicted N-terminal sequence accords most closely with the other staphylococcal DHFR proteins, but the actual DfrL protein may have an additional 17 amino acids at its N terminus (see Genbank Accession CAC8536249). Residues identical with DfrL are shown in black, with conserved residues in grey. An asterisk (*) indicates the position of the F_99_Y residue that is thought to underlie the trimethoprim resistance associated with DfrA, DrfG and DfrK. A circle indicates residues that are conserved across the DHFR family, including DfrL, but not in other staphylococcal DHFR enzymes.

We identified a novel diaminopyrimidine resistance determinant that has been designated *dfrL*. This gene was detected only once in our collection of strains, harboured by MOS222, a livestock-associated MRSA strain of clonal complex 398. The ancestral source of the *dfrL* gene is unknown, but there is evidence to suggest that it is not of recent staphylococcal origin. For example, DfrL contains residues (F_32_ and I_93_, numbering according to DfrB) that are near-universally conserved across the wider DHFR family but that are missing from all other staphylococcal representatives (Figure 4),^55^ and the GC content of *dfrL* is comparatively high; at 35.8%, it is higher than that of the plasmid on which it resides (31.7%) and GC content in the third codon position (GC3) is ∼30% (typical values for *S. aureus* genes are closer to ∼20%).^56^ The DfrL protein lacks the tyrosine residue at position 99 (Figure 4) that is considered to mediate resistance in the other staphylococcal trimethoprim-resistant DHFRs.^16^ Thus, the mechanism of resistance of DfrL appears distinct from these other enzymes, and further studies to understand this phenomenon are warranted. One potential explanation for the trimethoprim-resistant nature of DfrL is the presence of an isoleucine residue at position 93 in the active site, instead of the phenylalanine found in the trimethoprim-susceptible DfrB enzyme that appears to be important for binding trimethoprim.^16^

Clinical breakpoints defined by EUCAST and CLSI for a given drug may differ due to the distinct approaches each organization takes to determining such values.^57, 58^ We identified several instances of discordance when applying antifolate breakpoints for EUCAST and CLSI in this study, including (i) apparent susceptibility to co-trimoxazole in strains deemed—both by EUCAST and CLSI—to be independently resistant to both trimethoprim and sulfamethoxazole, (ii) resistance to co-trimoxazole according to CLSI only and (iii) resistance to co-trimoxazole (as per CLSI) while independently resistant to only one individual drug of the combination. Further studies would be required to establish which of the discordant parameters in each case accords most closely with successful therapeutic outcome. Harmonization of EUCAST and CLSI breakpoints would facilitate global reporting and surveillance of antifolate drug resistance in *S. aureus*.^59^

In conclusion, the prevalence of diaminopyrimidine resistance in our collection of MDR *S. aureus* is high, including to the investigational drug iclaprim. Further resistance surveillance is warranted for the latter if this agent is to be licensed. By contrast, resistance rates to co-trimoxazole appear comparatively low (∼4%) in MDR *S. aureus*, suggesting that resistance considerations should not preclude this combination drug from being considered for wider deployment in the management of staphylococcal infections.
